# Synthesis of Isotopically Labeled ^13^C_3_-Simazine and Development of a Simultaneous UPLC-MS/MS Method for the Analysis of Simazine in Soil

**DOI:** 10.3390/molecules21010089

**Published:** 2016-01-14

**Authors:** Yan Song, Yangzhen Guo, Xia Zhang, Yue Yang, Shuo Chen, Gaimei She, Dongmei She

**Affiliations:** 1 Plant Protection Institute of the Chinese Academy of Agricultural Sciences, Beijing 100193, China; songyan200714@163.com (Y.S.); guoyangzhen163@163.com (Y.G.); zhangxia0561@126.com (X.Z.); 18311242015@163.com (Y.Y.); chen@163.com (S.C.); 2 College of Pharmacology, Ningxia Medical University, Yinchuan 750004, China; 3 School of Chinese Pharmacy, Beijing University of Chinese Medicine, Beijing 100102, China; shegaimei@126.com

**Keywords:** ^13^C_3_-simazine, synthesis, IDMS, UPLC-MS/MS, isotope labelling

## Abstract

The isotope dilution mass spectrometry (IDMS) is a highly efficient method for tackling the ion suppression in complex matrix by ultra-performance liquid chromatography-tandem mass spectrometry (UPLC-MS/MS), but a lack of commercial internal standards is a limiting factor for these analyses. Herein, an economical and efficient strategy for the synthesis of ^13^C_3_-simazine via a three-step procedure was developed. The isotope-labeled internal standard was used for determination of simazine residue in soil samples. The quantitation method has a limit of detection of 0.015 μg/kg and quantitation of 0.08 μg/kg. The inter-day and intra-day precision of the method were below 4.6%. Recovery values were ranged between 92.9% and 99.2%. All the samples obtained from six provinces in China contained from 1 to 62 μg/kg of simazine.

## 1. Introduction

Simazine (6-chloro-*N*,*N*-diethyl-1,3,5-triazine-2,4-diamine) is a *s*-triazine herbicide which widely used for control of broadleaf weeds and annual grasses in corn, sugar cane and orchard. Owing its growth inhibitory effect on algal flora, the herbicide has been used in ornamental fish ponds, aquariums and fountains as an algicide [1]. Its mechanism of action involves selective inhibition of weeds’ photosynthesis via preventing electron transfer in the chloroplasts. However, recent researches showed that simazine could be toxic for the immune and reproductive system in animals [2,3,4,5,6,7,8,9], and further study revealed that it was probably an endocrine disrupter and potential carcinogen to human beings [10].

As a relatively difficult to degrade herbicide, simazine has been detected in many places. The EU Committee limited the use of simazine in 2007 due to its potential threat to human health. Meanwhile, America and Japan have implemented rigorous examination standards on the residue levels in drinking water. In 2006, the maximum contamination level of simazine in drinking water was limited to 4 ppb by the US EPA [11]. Therefore, an efficient and accurate method for the detection and quantification of trace residues in the environment is necessary.

In previous studies, the quantitative analyses of simazine include pre-column derivatization liquid chromatographic fluorescence detection (LC-FL) [12], antigen-coated tube enzyme-linked immune sorbent assay (ELISA) [13], high-performance liquid (HPLC) [14], gas chromatography coupled with mass spectrometric detection (GC-MS) [15] as well as liquid chromatography-tandem mass spectrometry (LC-MS/MS) [16,17,18]. Recently, LC-MS/MS has been widely applied in trace analysis of pesticides. Moreover, it is routinely used in food safety, pharmacokinetic experiments and clinical laboratories [19].

The matrix effect is the main problem for LC-MS/MS method quantification, and the inevitable matrix effect could normally reduce the method accuracy, precision and sensitivity, causing incorrect content determination [20]. As a result, isotope dilution mass spectrometry (IDMS) was developed to improve the situation [21]. During the analysis of samples, the isotope-labeled congeners were co-eluted reaching the mass spectrometer simultaneously and providing a consistent instrumental response. Hence, by measuring the isotopic ratio of the resulting sample solution via mass spectrometry, accurate analytical results can be procured. Generally speaking, the absence of commercially available isotope-labeled compounds limits the practical application of IDMS. The study reported herein aims to develop an effective and economical strategy for the synthesis of ^13^C_3_-simazine, and apply it as an internal standard for trace-level analysis of simazine in soil.

## 2. Results

### 2.1. Synthesis of ^13^C_3_-Simazine

^13^C_3_-simazine was synthesized from ^13^C-urea by three steps. Firstly, taking the stable isotope ^13^C-labeled urea as raw material, ^13^C_3_-cyanuric acid was synthesized using sulfolane-cyclohexanol as solvent. Next, ^13^C_3_-cyanuric chloride was prepared by chlorination with PCl_5_-POCl_3_. Finally, ^13^C_3_-simazine was obtained by reaction of ^13^C_3_-cyanuric chloride with ethylamine. The final product was characterized by FT-IR, ^1^H-NMR and MS. The ^1^H-NMR spectrum (CDCl_3_, 300 MHz) showed peaks at δ: 3.18–3.33 (m, 4H), 1.03–1.11 (m, 6H); and its molecular formula was determined as C_4_H_13_ClCt_3_N_5_ by the positive HRESIMS peak at *m*/*z* 205.09500 [M + H]^+^ (calcd. 205.09546). Its overall yield was 46.27%, the chemical purity was 99.00%, and the ^13^C abundance was 98.45%. It was qualified to be as an internal standard to detect the residue of simazine in soil.

### 2.2. Residue Analysis of Simazine in Soil

#### 2.2.1. Linearity, LOD and LOQ

The linearity of the calibration curves was evaluated using matrix matched calibration solutions with satisfactory linearity (y = 0.2730x + 0.1215, R^2^ > 0.9999). The ratio of slopes of calibration curves which obtained using the matrix-matched calibration solutions to that using the matrix-free counterpart was 0.97. It indicated that the internal standard ^13^C_3_-simazine could compensate the matrix effects in the UPLC-MS/MS chromatogram of a soil sample extract. The matrix-dependent LOD and LOQ values of the method were 0.015 and 0.08 μg/kg, respectively.

#### 2.2.2. Precision and Accuracy

A recovery study was carried out to evaluate the performance of the developed method. The spiking levels of blank samples were three concentrations (1, 5 and 10 μg/kg) with five replicates of each level. The precision of the method was determined by repeatability and reproducibility studies and expressed as the RSD. Recovery values were in the satisfactory rang of 92.9%–99.2% which were experimentally calculated through calibration curve. The intra-day RSD (*n* = 5) and inter-day RSD (*n* = 15) were in the range of 1.5%–4.6% and 2.1%–4.0%, respectively. Detailed recovery rates and repeatability dates are shown in Table 1.

**Table 1:** Relative recovery (%), intra-day RSD (%) and inter-day RSD (%) obtained for simazine in soil by UPLC-MS/MS.

Pesticide	Spiked Level (μg/kg)	Recovery (%)	Intra-Day RSD (%)	Inter-Day RSD (%)	LOQ (μg/kg)
Simazine	1	92.9	1.7	4.0	0.08
	5	97.7	4.6	3.9	
	10	99.2	1.5	2.1	

#### 2.2.3. Application to Actual Samples

The validated method was used to analyse soil samples. Among the ten samples, five were collected from five districts (Pinggu, Miyun, Shunyi, Tongzhou and Daxing) in Beijing. The process of extraction and purification is described in “Sample Preparation” below. The detection results of simazine residue are shown in Table 2.

**Table 2:** Concentrations of simazine residue in actual samples.

	**Pinggu**	**Miyun**	**Shunyi**	**Tongzhou**	**Daxing**
Simazine (μg/kg) ± RSD	62% ± 5.9%	49% ± 8.6%	61% ± 10.2%	43% ± 8.7%	14% ± 4.5%
	**Guangdong**	**Hainan**	**Hubei**	**Guangxi**	**Xiamen**
Simazine (μg/kg) ± RSD	2% ± 3.4%	1% ± 6.9%	15% ± 6.5%	19% ± 9.2%	13% ± 8.2%

## 3. Discussion

The UPLC-MS/MS method is a powerful technique for the determination of pesticide residues, but the accuracy and sensitivity of quantitative measurements are always affected by the sample matrix. According to the literature, when simazine was detected by LC-MS/MS without an isotope-labeled internal standard, the recovery values were about 60%–90% [22], but in this research, the values were significantly increased to 90%–100%. The result shows that the IDMS is an obligatory technique for achieving accurate quantification and high precision.

Even though many studies use deuterium-labeled congeners to detect the simazine residues [16,17], the ^13^C-labeled internal standard is more appropriate for accurate quantification, because its physico-chemical properties and chromatographic behavior are more similar to the native target analysed [23].

In our research, an economical and efficient strategy for the synthesis of ^13^C_3_-simazine was developed. During the synthesis process, a previous synthesis of the similar compound ^13^C_3_-atrazine was used as a guide [24]. At the first step, ^13^C_3_-cyanuric acid was produced from ^13^C-urea through thermolysis and condensation at 200 °C. The mixture of sulfolane-cyclohexanol was applied as medium, which contributed to dissolving the urea and precipitating the product, which could effectively prevent the generation of amine derivatives. In addition, the synthesis system should be placed under vacuum which helps reduce the concentration of ammonia. All of these steps help to get a higher yield. In the second step, the ^13^C_3_-cyanuric chloride was prepared by a chlorination reaction. The reagent PCl_5_-POCl_3_ is a good provider of chlorine for *s*-triazine derivatives [25,26,27]. Meanwhile, the reaction was finished in one reactor, which could reduce the waste of raw materials and product in the process. Finally, the conversion of ^13^C_3_-cyanuric chloride to ^13^C_3_-simazine was quite straightforward. This synthesis method is reported for the first time in this study.

Furthermore, the synthesized ^13^C_3_-simazine was applied to detect actual samples. The results showed that pollution in Beijing was far above than in the other five provinces. Meanwhile, Guangdong and Hainan’s soil contents are lower in simazine, suggesting that environmental differences maybe the key reasons. In the south of China, the temperature is higher and the soil has less organic matter content, and in addition the application rates used are relatively lower. The higher temperature could enhance the activity of microorganisms to accelerate the decomposition of simazine.

## 4. Experimental Section

### 4.1. Regents and Standards

All organic solvents used to synthesis of ^13^C_3_-simazine were analytical grade reagents. ^13^C-Urea (99 atom% ^13^C) was purchased from Sigma-Aldrich (Steinheim, Germany). KBr was obtained from Thermo-Fisher Scientific (Waltham, MA, USA). An analytical standard of simazine (99.5% purity) were purchased from AccuStandard (New Haven, CT, USA). Liquid chromatography (LC)-grade acetonitrile and methanol were purchased from Sigma-Aldrich. Ultrapure water was prepared using a Milli-Q reagent water system (Millipore, Bedford, MA, USA). Anhydrous magnesium sulfate (MgSO_4_), sodium chloride (NaCl) and analytical grade methanol were purchased from Beijing Chemical Company (Beijing, China). PSA (40 μm) was obtained from Agela Technologies (Beijing, China). The purchased certified analytical standard of simazine (1 mg) was dissolved in 1000 mL of LC-grade methanol, which resulted in a stock solution (1000 μg/L). The ^13^C_3_-simazine stock solution (1000 μg/L) was prepared according to the same method. The working solutions of simazine (100 μg/L) and the ^13^C_3_-simazine (100 μg/L) were prepared in LC-grade methanol for use as spiking solution and for the analytical curves. All solutions were stored at −20 °C under exclusion of light to ensure stability.

### 4.2. Soil Samples

Soil samples were collected in five regions of Beijing and another five Chinese provinces from agricultural areas where simazine is widely used. Herbicide-free soil samples were collected from a vacant area of the Plant Protection Institute of the Chinese Academy of Agricultural Sciences (Beijing, China). They were dried at room temperature and homogenized through 1-mm sieve. The characteristics of the soil samples used in this study were a pH of 5–8 and 0.87%–3.25% organic matter. The blank samples were confirmed to be free of simazine residues by UPLC-MS/MS analysis.

### 4.3. Instrumentation

Infrared spectra were recorded in KBr pellets on a 6700 FT-IR spectrophotometer (Thermo Nicolet, New Orleans, LA, USA). The ^1^H-NMR (300 MHz) spectra were recorded on a Bruker DPX 300 MHz NMR spectrometer (Bruker Corporation, New Orleans, LA, USA) and chemical shifts values were given in parts per million relative to TMS in DMSO-*d*_6_. HRMS was performed on a Bruker Apex IV FTMS (Bruker Corporation). Chromatographic separation was performed with an Acquity UPLC system (Waters, Milford, MA, USA). The analytical column was Acquity UPLC BEH C18 (2.1 mm × 100 mm, 1.7 μm) from Waters. The mobile phase was composed of solvent A (acetonitrile) and solvent B (0.2% formic acid in water) at a constant flow of 0.3 mL/min. Elution was performed in the gradient mode (0 min, 30% solvent A; 1.5 min, 90% solvent A; 3.1 min, 30% solvent A; 5.0 min, 30% solvent A), and the total analysis time was 5 min. The temperatures of the column oven and the sample manager were set at 45 °C and 15 °C, respectively. Detection was achieved using a triple quadrupole mass spectrometer (Waters) fitted with an electrospray ionization (ESI) source, operating in positive mode, and controlled by MassLynx software. The source parameters was optimized as follows: capillary voltage, 3.02 kV; source temperature, 148 °C; desolvation temperature, 350 °C; desolvation gas (N_2_) flow rate, 650 L/h; cone gas (N_2_) flow rate, 50 L/h. The multiple-reaction monitoring transitions, cone voltage and collision energy were optimized to reach highest sensitivity and resolution, which the parameters is showed in Table 3.

**Table 3:** Tandem mass spectrometry parameters used for analysis of simazine.

Compound	CV (V)	Quantification Ion Transition	CE1 (eV)	Confirmatory Ion Transition	CE2 (eV)	Ion Ratio
Simazine	32	202 → 132	21	202 → 124	19	0.82
^13^C_3_-Simazine	30	205 → 134	24	205 → 126	20	0.75

CE: collision energy; CV: cone voltage; Ion ration = area of qualitative ion/area of quantification ion.

### 4.4. Synthesis of ^13^C_3_-Simazine

#### 4.4.1. Synthesis of ^13^C_3_-Cyanuric Acid

**Scheme 1:**
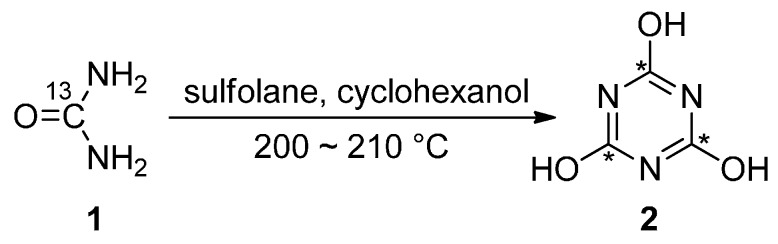
^13^C_3_-Cyanuric acid synthesized from ^13^C-urea.

A mixture of sulfolane (12 g, 0.100 mol) and cyclohexanol (4 g, 0.040 mol) was stirred in a 50 mL three-neck flask equipped with a reflux condenser. When the temperature reached 60 °C, ^13^C-urea (4 g, 0.067 mol) was added into the flask. Under negative pressure, the reaction mixture was refluxed for 3 h at 200–210 °C until the system did not generate ammonia any longer. The reaction system was cooled 50 °C, and the precipitate was filtered and washed with ethyl alcohol. The final product was dried in an oven at 60 °C for 2 h. A total of 2.5 g (0.019 mol) of crude product was obtained, and the yield was 81.8% (Scheme 1).

#### 4.4.2. Synthesis of ^13^C_3_-Cyanuric Chloride

**Scheme 2:**
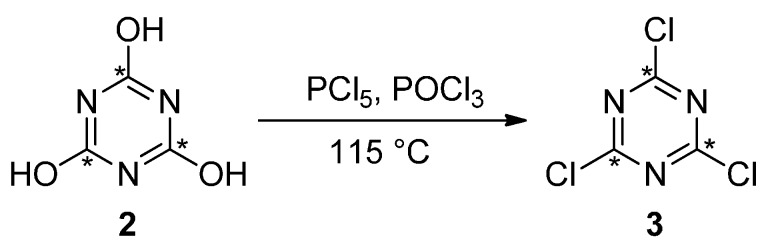
^13^C_3_-Cyanuric chloride synthesized from ^13^C_3_-cyanuric acid.

^13^C_3_-cyanuric acid (0.90 g, 0.007 mol), phosphorus pentachloride (4.37 g, 0.021 mol) and phosphorus oxychloride (3.22 g, 0.021 mol) were added to a three-neck-flask and stirred and refluxed at 115 °C for 24 h until no more HCl was released. Finally, the residue was distilled at 120 °C under vacuum and the phosphorus oxychloride was removed. The product was washed with chloroform from the inside of the condenser, and the solvent was evaporated under reduced pressure. A white solid (0.94 g, 0.005 mol) was obtained. The yield in this step was 73.2% (Scheme 2).

#### 4.4.3. Synthesis of ^13^C_3_-Cyanuric Chloride

**Scheme 3:**
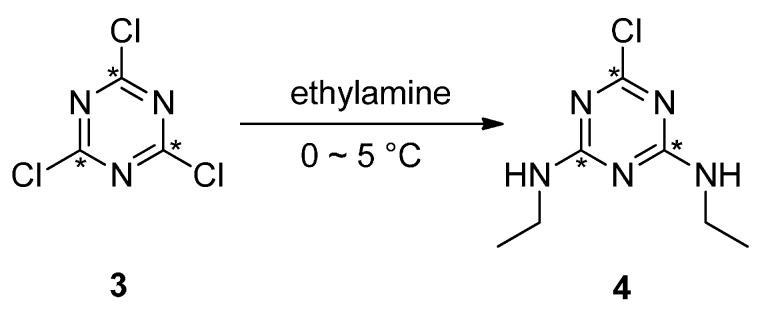
^13^C_3_-Simazine synthesized from ^13^C_3_-cyanuric chloride.

A mixture of ^13^C_3_-cyanuric chloride (4.07 g, 0.022 mol) and chloroform (20 g) was placed in a three-neck-flask. After cooling down to 0 °C, ethylamine (4.5 g) was slowly added dropwise into the mixture that was stirred at 0–5 °C for 2 h. After filtering and washing with methyl alcohol (30 mL) the simazine was dried under vacuum at 60 °C for 2 h. A total of 3.42 g (0.017 mol) of product was obtained, for a yield of 77.27% (Scheme 3).

### 4.5. Residue Analysis of Simazine in Soil

#### 4.5.1. Sample Preparation

The modified QuEChERS method was employed in the extraction of soil samples. Soil samples (10 g) were weighted into 50 mL polypropylene centrifuge tubes. The internal standard, ^13^C_3_-simazine, was added to reach a target level of 10 μg/kg. Then, methanol (10 mL) was added and the mixture was vortexed for 5 min. Next, NaCl (2 g) and MgSO_4_ (3 g) were added into the tubes. After shaking for 5 min, samples were centrifuged for 5 min at 4000 rpm. Following that, a portion (1.5 mL) of the upper methanol layer was transferred into a 2 mL centrifuge tube containing PSA (50 mg) and MgSO_4_ (150 mg). The mixtures were vortexed for 1 min and centrifuged for 5 min at 4000 rpm. Finally, the supernatant was filtered through 0.22 μm nylon syringe filter and stored at −20 °C in sealed containers until analysis.

#### 4.5.2. Method Validation

The method validation was evaluated on the basis of following parameters: calibration cure and linearity, limit of detection (LOD), limit of quantification (LOQ), accuracy (in terms of recovery), and precision (in terms of repeatability and intermediate precision). The calibration curves were plotted by the peak area ratios of the simazine and the ^13^C_3_-simazine against the corresponding concentration ratios. A total of seven concentrations points at 1, 2, 5, 10, 20, 50 and 100 μg/L were prepared in triplicate, with 10 μg/L ^13^C_3_-simazine in each solution. The matrix standard solutions were prepared by blank sample of soil according to the sample preparation. The LOD and LOQ of method were also determined by matrix standard solutions. The values were evaluated using the 3 and 10 times the ratio of signal-to-noise, respectively, corresponding to the lowest point used in the matrix-assisted calibration. 

The accuracy and precision of the method were evaluated by analysis of blank samples spiked at three levels. 10 g blank soil samples were spiked with 1, 5 and 10 μg/kg, and the spiking level of ^13^C_3_-simazine was kept at 10 μg/kg. The spiked samples were blended and left to stand for 30 min. After this, samples were extracted and purified based on aforementioned method. There were five replicates for every spiking level and these were conducted on three different days. The precision was same as repeatability in these conditions. It was expressed through the relative standard deviation (RSD) and evaluated by the intra-day and inter-day precision.

## 5. Conclusions

In summary, we have successfully synthesized stable isotope labeled ^13^C_3_-simazine with excellent yield and high purity. The stable isotope ^13^C was introduced into three sites of simazine. The low-cost ^13^C-urea used as raw material is economical. The chemical purity of the synthesized ^13^C_3_-simazine was more than 99%, and the ^13^C abundance was beyond 98% and it was qualified to be as internal standard. 

Simazine residues of soil were determined by UPLC-MS/MS based on a modified QuEChERS extraction method. The IDMS provided more reliable analytical values compared with the conventional external calibration method. A low detection limit with satisfactory recoveries and good precision were achieved in the validation procedure. In all the tested areas (five districts in Beijing, Guangdong, Hainan, Hubei, Guangxi and Xiamen, simazine residues were found, so future detection work conducted on soil should draw more attention to the presence of simazine residues.

## Supplementary Materials

Supplementary materials can be accessed at: http://www.mdpi.com/1420-3049/21/1/89/s1.
