# Supplementary material for: Synthesis of Isotopically Labeled 13C3-Simazine and Development of a Simultaneous UPLC-MS/MS Method for the Analysis of Simazine in Soil
Source: Molecules. 2016 Jan 14;21(1):89. doi: 10.3390/molecules21010089 (PMC6273493; doi:10.3390/molecules21010089)
Supplement: Supplementary file 1 [file molecules-21-00089-s001.pdf]

Supplementary Materials: Synthesis of Isotopically Labeled $^{13}C_3$-Simazine and Development of Simultaneous UPLC-MS/MS for the Analysis of Simazine in Soil

Yan Song [1,2], Yangzhen Guo [1], Xia Zhang [1,3], Yue Yang [1,3], Shuo Chen [1], Gaimei She [3] and Dongmei She [1,*]

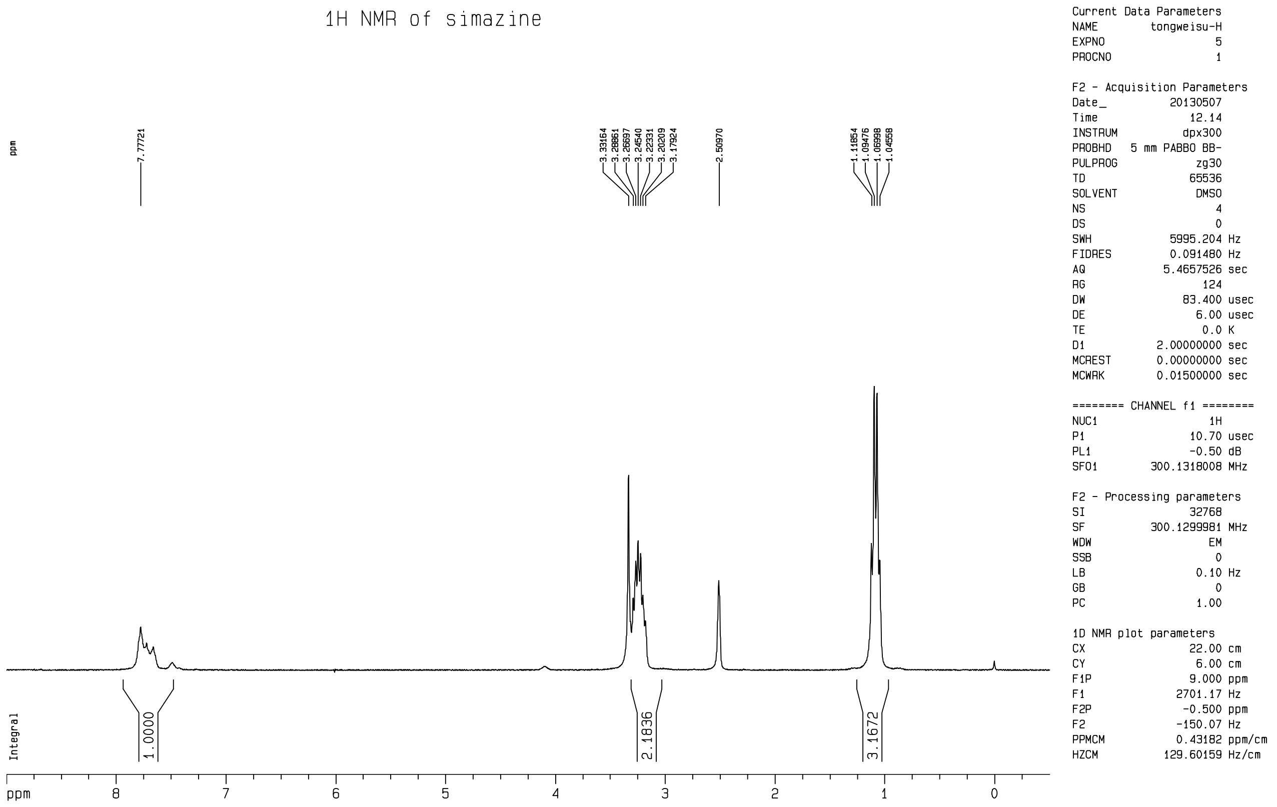

Figure 1. The ^{1}H-NMR spectrum of $^{13}C_3$-labelled simazine.

Analysis Info

Analysis Name	13060648_20130618_000001.d	Acquisition Date	6/18/2013 11:12:45 AM
Sample	Simazine-C13	Instrument	Bruker Apex IV FTMS
Comment	ESI Positive	Operator	Peking University

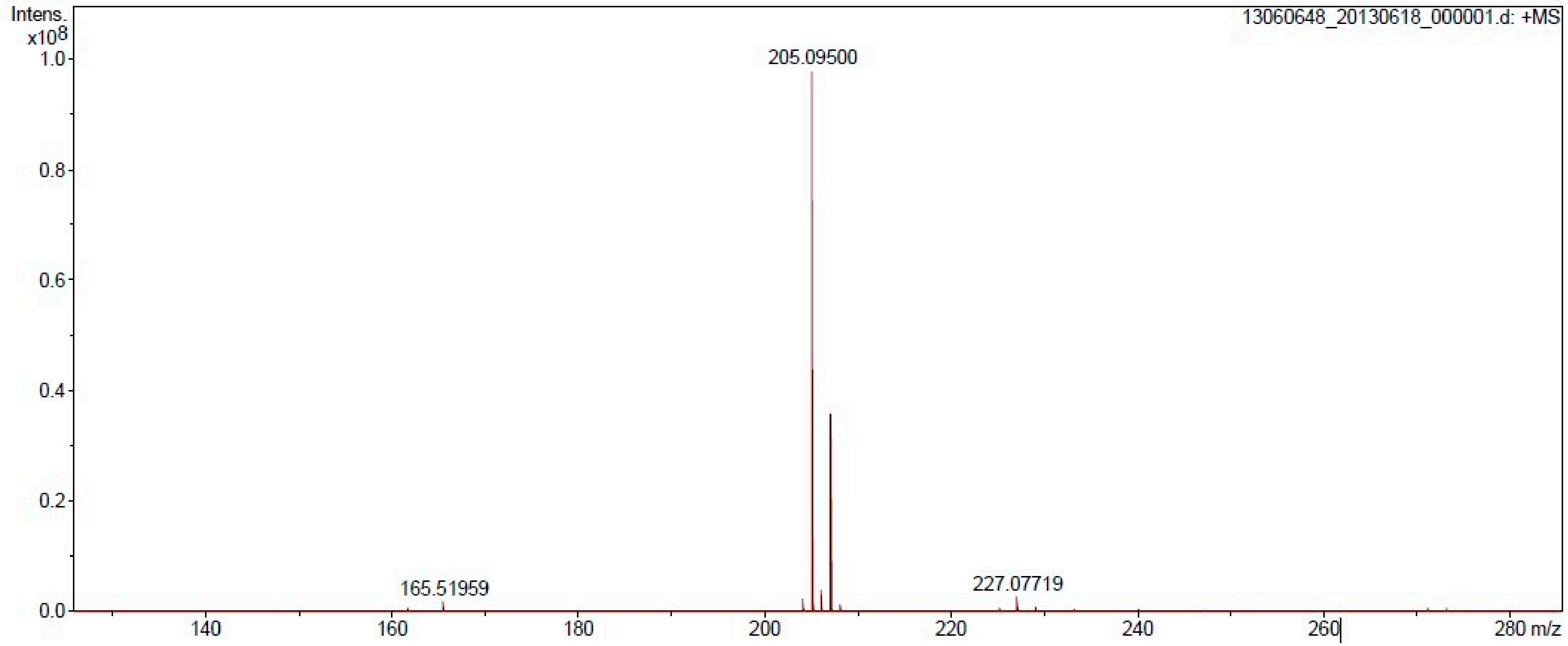

Meas. m/z	#	Formula	Score	m/z	err [mDa]	err [ppm]	mSigma	rdb	e⁻ Conf	N-Rule
205.09500	1	C 4 H 13 Cl Ct 3 N 5	100.00	205.09546	0.5	2.3	74.1	3.5	even	-

Figure 2. The MS spectrum of $^{13}C_3$-labelled simazine.

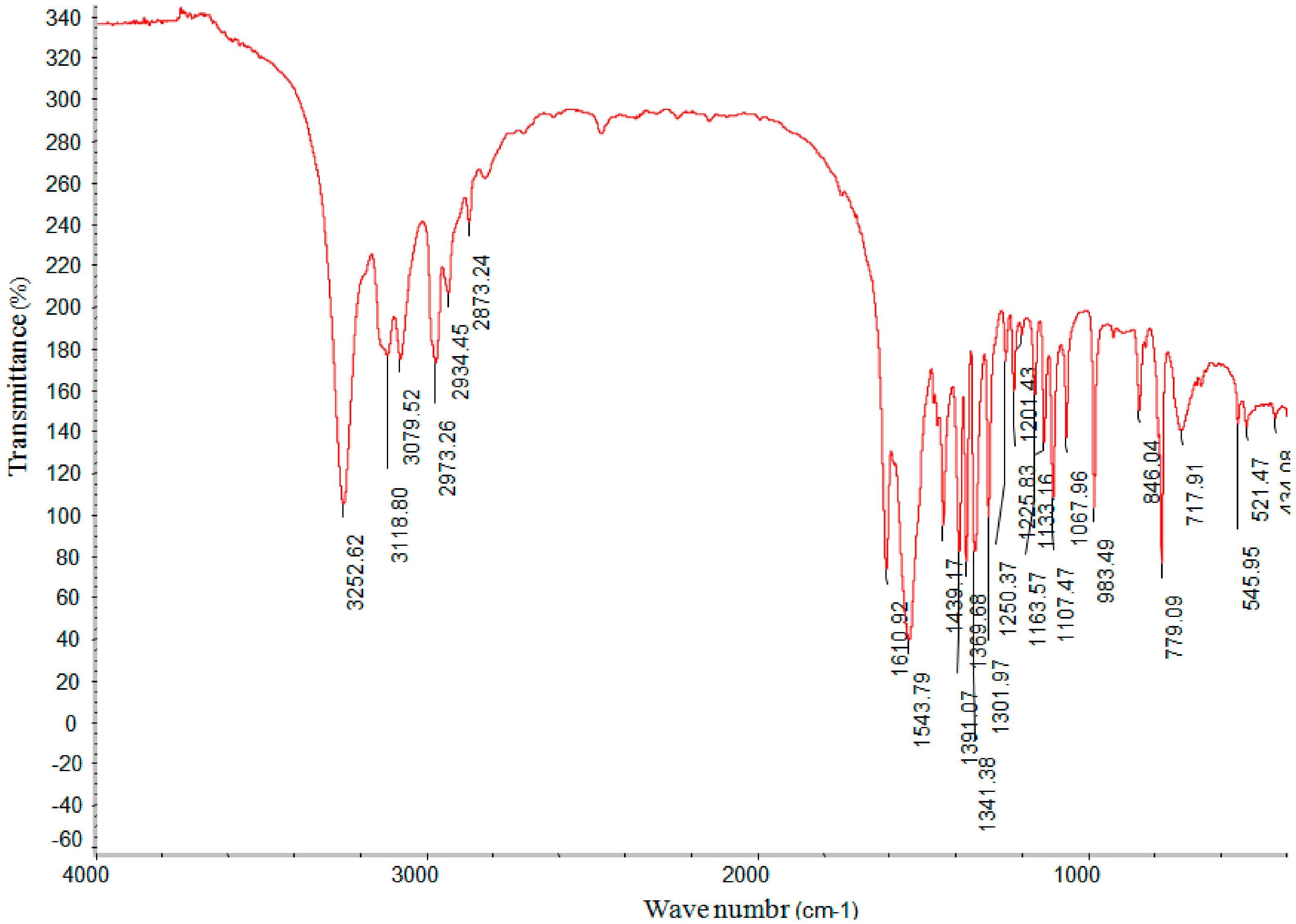

Figure 3. The IR (Thermo Nicolet 6700 FT-IR) spectrum of $^{13}C_3$-labelled simazine.

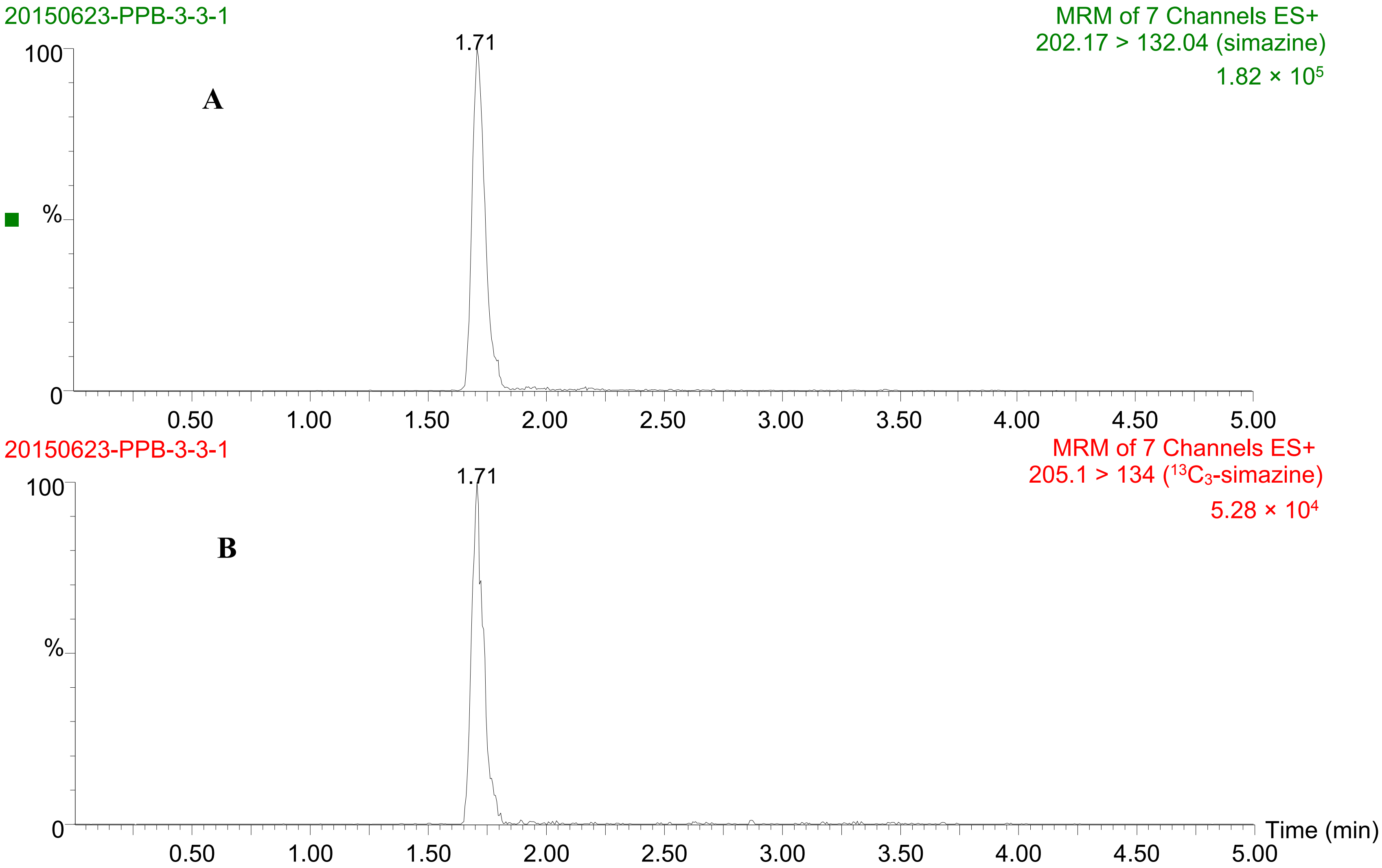

Figure 4. Positive ESI-HPLC-MS/MS chromatograms of an extract of a soil sample, (**A**) simazine and (**B**) $^{13}C_3$-simazine.
